# Prevalence of Juvenile-Onset and Pediatric Huntington’s Disease and Their Availability and Ability to Participate in Trials: A Dutch Population and Enroll-HD Observational Study

**DOI:** 10.3233/JHD-240034

**Published:** 2024-09-10

**Authors:** Hannah S. Bakels, Stephanie Feleus, Mar Rodríguez-Girondo, Monique Losekoot, Emilia K. Bijlsma, Raymund A.C. Roos, Susanne T. de Bot

**Affiliations:** a Department of Neurology, Leiden University Medical Centre, Leiden, The Netherlands; b Department of Clinical Epidemiology, Leiden University Medical Centre, Leiden, The Netherlands; c Department of Biomedical Data Sciences, Leiden University Medical Centre, Leiden, The Netherlands; d Department of Clinical Genetics, Leiden University Medical Centre, Leiden, The Netherlands

**Keywords:** Juvenile-onset Huntington’s disease, pediatric Huntington’s disease, prevalence, incidence, trial eligibility, disease stage

## Abstract

**Background:**

Juvenile-onset Huntington’s disease (JHD) represents 1–5% of Huntington’s disease (HD) patients, with onset before the age of 21. Pediatric HD (PHD) relates to a proportion of JHD patients that is still under 18 years of age. So far, both populations have been excluded from interventional trials.

**Objective:**

Describe the prevalence and incidence of JHD and PHD in the Netherlands and explore their ability to participate in interventional trials.

**Methods:**

The prevalence and incidence of PHD and JHD patients in the Netherlands were analyzed. In addition, we explored proportions of JHD patients diagnosed at pediatric versus adult age, their diagnostic delay, and functional and modelled (CAP^100^) disease stage in JHD and adult-onset HD patients at diagnosis.

**Results:**

The prevalence of JHD and PHD relative to the total manifest HD population in January 2024 was between 0.84–1.25% and 0.09–0.14% respectively. The mean incidence of JHD patients being diagnosed was between 0.85–1.28 per 1000 patient years and of PHD 0.14 per 1.000.000 under-aged person years. 55% of JHD cases received a clinical diagnosis on adult age. At diagnosis, the majority of JHD patients was functionally compromised and adolescent-onset JHD patients were significantly less independent compared to adult-onset HD patients.

**Conclusions:**

In the Netherlands, the epidemiology of JHD and PHD is lower than previously suggested. More than half of JHD cases are not eligible for trials in the PHD population. Furthermore, higher functional dependency in JHD patients influences their ability to participate in trials. Lastly, certain UHDRS functional assessments and the CAP^100^ score do not seem appropriate for this particular group.

## INTRODUCTION

Juvenile-onset Huntington’s disease (JHD) represents a small group of Huntington’s disease (HD) patients with motor disease onset≤20 years of age. JHD patients can be subdivided in childhood-onset JHD (cJHD; onset between 0–10 years) and adolescent-onset JHD (aJHD; onset between 11 and 20 years).^1,2^ The age at disease onset in HD is negatively correlated with the causal number of CAG-repeats in in the Huntingtin (HTT) gene (≥36), explaining approximately 60% of variability in adult-onset HD (AHD) and up to 84% in JHD.^3^ Approximately 50% of JHD cases have a CAG≥60, even exceeding 80 CAGs in rare cJHD cases.^4^ Although there are JHD cases reported with CAG-repeats in the lower abnormal CAG-range (CAG 40–50),^5^ the likelihood of developing a juvenile phenotype exceeds 5% in case of a CAG≥51.^6^ JHD patients are thought to represent approximately 1–5% of the total number of clinically manifest HD patients.^7,8^ This, together with an estimated mean prevalence of 4–6 clinical HD patients per 100.000 in the Western European population,^9–11^ indicates that the number of JHD patients is very low. The majority of JHD patients represents aJHD, with an estimated proportion of 4.4%, and as little as 1.3% represents cJHD patients.^7^

Over the years a variety of studies, reviews and meta-analyses reported the epidemiology of (J)HD, which is subject to constant change such as earlier recognition and diagnosis. One recent development that relies on the number of JHD patients is the removal of the European Medical Agency (EMA) class waiver for pediatric patients in the HD population, dictating a pediatric investigation plan for the study of new therapeutic strategies.^12^ Pediatric HD (PHD) refers to a proportion of JHD patients that is below the age of 18 years, as opposed to JHD patients that became clinically manifest before that age but that have grown into adulthood (≥18 years).^12^ Up to now there has been one study analyzing the number of PHD patients in the international ENROLL-HD dataset, which found proportional margins between 0.14–0.66% of the total number of manifest HD patients.^8^ These numbers are even lower than may be expected from earlier epidemiology studies in the JHD population.^7^ Accurate numbers of the current prevalence and incidence of the JHD population, as well as the proportion of JHD patients being diagnosed as a minor or adult, is important when it concerns the design of interventional trials in JHD or PHD patients.

Another important question is the ability of the JHD population to participate in interventional studies. Part of cJHD patients are known to have a faster disease progression and with a shorter survival compared to AHD.^1^ Together with the diagnostic delay in the pediatric population,^13–15^ this may substantially influence the disease stage in which JHD patients reside when they are diagnosed and their availability to participate in interventional trials. Insight in the correlation between diagnosis on the one hand and disease stage markers, such as functional competence and by the normalized predictor CAG-Age-Product (CAP^100^) score,^16^ on the other hand, helps defining the ability of the JHD population to actually participate in interventional trials.

The aim of our study is to describe the current prevalence of PHD and JHD patients in the Netherlands relative to the entire manifest HD population and to determine 5-year incidences of the Dutch JHD and PHD population over the past 20 years. Furthermore, the availability and ability of the JHD population, to participate in interventional trials, is analyzed by 1) the proportion of JHD patients being diagnosed at a pediatric vs. adult age and 2) comparing disease stage markers at the time of diagnosis between JHD and AHD patients.

## MATERIALS AND METHODS

### Study design

Data from two (J)HD patient datasets was used to answer the different study goals and to enable comparison of JHD with prototypical disease onset in adulthood (AHD). The first, HD-JUNIOR, is a multi-source Dutch registry for JHD patients. HD-JUNIOR began in 2020 and consists of the following complementary datasets: 1) pseudonymized demographic and HTT genetic data from all HD expanded gene carriers with a CAG≥51 (*n* = 121) that were tested in the Netherlands since 2000 and that is annually updated, combined with 2) retrospective clinical data from medical files of clinically diagnosed JHD patients (irrespective of HTT genetic status) that were derived from all HD care facilities in the Netherlands and additional medical sites by pearl-growing method (*n* = 28). For this study, only cases were included where clinical data showed that the patient had a JHD phenotype. Written informed consent for the collection and use of pseudonymized clinical data was given by all living JHD patients or their caretakers. In the case of clinical data from deceased JHD patients, pseudonymized data was shared by the last treating physician. The second dataset, ENROLL-HD, is an international prospective longitudinal registry study in HD expanded gene carriers (≥36 CAG-repeats) and controls.^17^ For the current study, the 5th periodic dataset (PDS5; release 18-DEC-2020; *n* = 21,116 participants) was used to retrieve genetic and clinical data from JHD and AHD patients, including a specified dataset with deaggregated data for age at enrolment below 17 years and number of CAG-repeats≥70. Data were generously provided by the participants in the Enroll-HD study and made available by CHDI Foundation, Inc. Core datasets were collected annually from all research participants as part of this multi-center longitudinal observational study. Data were monitored for quality and accuracy using a risk-based monitoring approach. All sites were required to obtain and maintain local ethical approval. In case of outcome measures with a similar assessment method in both datasets, the results for the JHD subtypes of the two different datasets were pooled provided that the baseline JHD sample characteristics of the two datasets were comparable (in total: cJHD *n* = 44; aJHD *n* = 120; AHD *n* = 8808). Duplicate cases in the two different datasets were identified by the combination of CAG-repeat length and year of birth and corrected for in case of pooled analyses.

### Study population

**Fig. 1:**
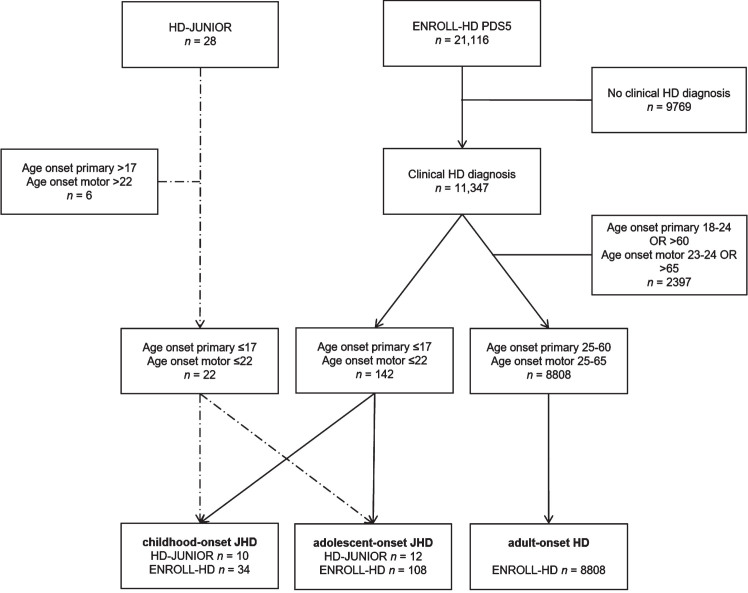
Patient selection of the HD-JUNIOR and ENROLL-HD PDS5 datasets. STROBE flow diagram displaying patient selection of the HD-JUNIOR and ENROLL-HD PDS5 datasets based on the eligibility criteria for the current study and stratified by AO-HD subtype. Dashed lines represent patient selection from the HD-JUNIOR dataset into the two different JHD subtypes, straight lines represent patient selection from the ENROLL-HD PDS5 dataset into the three different AO-HD subtypes.

This study uses below defined age at onset-defined HD (AO-HD) subtypes, clinically manifest disease status and current age as grouping variables. Based on a lower prevalence of motor disease characteristics at onset in JHD patients,^18^ we chose to define a JHD phenotype primarily on the basis of age at onset of any HD symptom or sign (e.g., psychiatric, neurocognitive, motor or neurodevelopmental) and subsequently on age at onset of motor symptoms, which had to occur within 5 years of first symptoms. Inclusion criteria for this study were as follows: 1) a clinical diagnosis of HD (Unified Huntington Disease Rating Scale – Total Motor Score: Disease Confidence Level of 4 →≥99% confidence motor abnormalities are unequivocal signs of disease) based on expert opinion and irrespective of CAG-repeat length, 2) onset of first symptom≤17 years of age, and 3) onset of motor symptoms≤22 years of age. Subsequently, JHD patients were subdivided in childhood-onset JHD (cJHD: primary onset≤10 and motor onset≤15 years of age) and adolescent-onset JHD phenotype (aJHD: primary onset between 11 and 17 and motor onset between 11 and 22 years of age, or a primary onset≤10 and motor onset between 16 and 22 years of age). Definition of PHD was 1) a clinical diagnosis of HD based on expert opinion and irrespective of CAG-repeat length, 2) onset of first and motor symptoms≤17 years of age, and 3) current age≤17 years. For the comparison of disease stage markers in JHD subtypes with prototypical disease onset in adulthood, eligibility criteria for an AHD phenotype in this study were based on an age at primary and or motor onset≥25 and≤60 (primary onset) /≤65 (motor onset) years of age, to ensure that there is no overlap in disease phenotypes and to limit the influence of aging effects. See STROBE- flow diagrams for the number of eligible AO-HD defined cases in the HD-JUNIOR and ENROLL-HD datasets (Fig. 1).

### Outcome measures

To determine the point prevalence of the JHD and PHD population in the Netherlands, we defined the number of alive JHD and PHD individuals in the HD-JUNIOR registry at 1-JAN-2024. HD-JUNIOR is a national registry containing all genetic and demographic data (including survival) from HD expanded gene carriers with a CAG≥51 in the Netherlands, as well as verified JHD clinical data (irrespective of CAG-repeat length) retrieved from over 20 sources (find full credentials under acknowledgements), including all specialized HD care facilities in the Netherlands, and general practitioners, revalidation institutions, regional and academic medical centers by Pearl-growing method. The non-invasive nature of HD-JUNIOR (retrospective data collection, no extra assessments, informed consent procedure via phone/e-mail) ensures a very low-threshold to participate, even for JHD patients in later disease stages. Therefore, this comprehensive multi-source dataset gives a reliable estimate of epidemiological counts of the JHD and PHD population in the Netherlands. To calculate the relative proportion of these prevalent JHD and PHD cases as part of the entire clinically manifest HD population in the Netherlands, an estimated mean prevalence of 4–6:100,000 in Western Europe,^9–11^ and a total population of 17,947,684 in the Netherlands at 31-DEC-2023 were used. In addition, frequencies between 2000 and 2019 were determined for JHD patients 1) developing primary symptom onset, 2) developing motor symptom onset, 3) receiving a diagnosis, and 4) who deceased. Mean incidences of JHD diagnosis and JHD patient death were placed into perspective by the estimated total manifest HD population in the Netherlands in the same time period. In addition, the mean incidence rate of a PHD diagnosis was placed into perspective by the total under-aged (≤17 years) general population in the Netherlands in the same time period. Incidence rates were compared in 4 consecutive time intervals: from 2000 through 2004; from 2005 through 2009; from 2010 through 2014; and from 2015 through 2019.

For the definition of age and disease duration at diagnosis we used data from the HD-JUNIOR and ENROLL-HD dataset. Both datasets contain retrospectively collected data specifying the age or year at which a certain individual experienced the first symptom or received his/her clinical diagnosis based on expert opinion. We used age to dichotomize between patients receiving their diagnosis on pediatric age (≤17 years) and patients receiving their diagnosis on adult age (≥18 years). For individuals of the HD-JUNIOR dataset, the number of JHD patients that received their genetic status via preclinical genetic testing was additionally specified. Because ‘preclinical’ genetic testing in the Netherlands is only available for adult HD expanded-gene at risk individuals, these JHD cases received their genetic status prior to receiving a clinical diagnosis, but both on an adult age, and were labeled as JHD in retrospect. Data regarding preclinical genetic testing in the PDS5 of ENROLL-HD were not available. To compare AO-HD subtypes on diagnostic delay, we used disease duration between first symptom and clinical HD diagnosis, as captured retrospectively in both datasets.

To analyze the ability of JHD patients to participate in clinical trials, disease stage markers at the time of diagnosis were explored and, where possible, compared with those of AHD patients at diagnosis. The first, functional capacity, is a common measure to reflect the severity or disease stage in clinically manifest HD.^19–21^ HD-JUNIOR data relating to functional capacity at diagnosis in JHD patients, were retrieved retrospectively from multi-source medical files carrying unspecified data from anamnesis or care taker reports. For the current study, we used all data that indicated a decline in skills, the need for help or the need to give up previously established activities such as education or work (cJHD *n* = 5; aJHD *n* = 8). To assess functional capacity in the ENROLL-HD dataset, prospective data from the Unified Huntington Disease Rating Scale– Independence Score (UHDRS-IS) ^22^ in aJHD (*n* = 33) and AHD (*n* = 3186) participants was used and compared, in case this data was captured within one year of receiving a clinical HD diagnosis. Because only four cJHD participants in ENROLL-HD had these data available within one year of diagnosis, these data were only described but not included in AO-HD subtype comparison. We deliberately chose not to include functional measures that were designed for adult participants. Particularly the UHDRS– Total Functional Capacity (UHDRS-TFC) and part of the UHDRS-Functional Assessment Scale (UHDRS-FAS) are not suited for pediatric participants as it focuses on outcomes that are generally not applicable to the pediatric population, such as working ability, finances and doing domestic chores.^22^ A second disease stage measure used was the CAG-Age Product (CAP) formula that is commonly used as a predictor for HD disease progression and reflects the cumulative exposure to the effects of mutant huntingtin by the interaction of CAG-repeat length and age. For the current study we calculated the CAP^100^ score,^16^ at 1) primary symptom onset, 2) motor symptom onset, and 3) HD clinical diagnosis by the formula: AGE * (CAG - 30) / 6.49. This CAP formula is normalized for CAG-repeat lengths up to 50, so that the CAP score approximates 100 when HD patients generally receive their clinical diagnosis, henceCAP.^100^

### Statistics

IBM SPSS Statistics version 29.0.0.0 (241) was used for statistical analyses.

Outcome measures and patient characteristics were described using mean and standard deviation if they were approximately normally distributed or median and interquartile range (IQR) otherwise. Prevalence frequencies and proportions were calculated and 95% Confidence Intervals (CI) for proportions were calculated. Frequencies, mean and 95% CI were calculated to determine the incidence rate of JHD diagnosis and death in relation to the total clinically manifest HD population and the incidence rate of PHD diagnosis in relation to the total under-aged general population in the Netherlands (PHD). 95% CI for the means of normally distributed data was used in case of the CAP^100^ score.

For between group comparison of the incidence, disease duration, UHDRS-IS and CAP^100^ outcome measures one-way ANOVA was performed and *p*-values < 0.05 were considered significant. In case of multiple testing, 95% CI and *p*-values were adjusted for multiple testing by Benjamini Hochberg method. In case of non-normal distribution, the outcome measure was log10-transformed. This was done for disease duration and UHDRS-IS score.

## RESULTS

### Patient characteristics

The number of eligible subjects in the HD-JUNIOR and ENROLL-HD dataset and stratified by AO-HD subtype are provided in Table 1. The included patient characteristics for sex, age at onset of primary and motor symptoms and CAG-repeat length did not significantly differ between the datasets. Therefore, results for JHD subtypes from the two different datasets were pooled in case of a comparable measurement method. These measures included disease duration at diagnosis and CAP^100^ score over time.

**Table 1:** Patient characteristics per AO-HD subtype and dataset

	Childhood-onset JHD			Adolescent-onset JHD			Adult-onset HD
	HD-JUNIOR	ENROLL-HD	*p*	HD-JUNIOR	ENROLL-HD	*p*	ENROLL-HD
	(*n* = 10)	(*n* = 34)		(*n* = 12)	(*n* = 108)		(*n* = 8808)
Sex, M/F %	50/50	47/53	0.870	58/42	50/50	0.584	48/52
Age onset primary symptom, Mean±SD [Range]	6±2 [4–10]	6±2 [2–10]	0.835	15±2 [12–17]	15±2 [11–17]	0.421	44±9 [25–60]
Age onset motor symptom, Mean±SD [Range]	7±3 [4–11]	8±4 [1–15]	0.767	17±2 [13–20]	16±3 [11–22]	0.638	45±9 [25–65]
CAG-repeat, Mean±SD [Range]	74±12 [52–92]	75±17 [48–110]	0.787	59±5 [51–66]	60±8 [43–81]	0.579	44±3 [36–62]

*n,* number of patients; SD, standard deviation; M, male; F, female; CAG-repeat, Cytosine-Adenine-Guanine repeats in the Huntingtin gene.

### Prevalence of JHD in the Netherlands

On January 1, 2024, there were 9 living JHD cases fulfilling the eligibility criteria for JHD in the HD-JUNIOR registry (Table 2). Six cases had an adolescent-onset JHD phenotype, 3 cases a childhood-onset JHD phenotype. Of these 9 cases, 1 was still under the age of 18 years, therefore referred to as PHD. Based on a clinically manifest HD prevalence estimate of 4–6:100,000 and a Dutch population of 17,947,684 on December 31, 2023, the estimated absolute number of the total clinically manifest HD population in the Netherlands was between 718 and 1077 cases. The prevalence of JHD as a percentage of the total clinically manifest HD population was between 0.84 to 1.25% (95% CI 0.29–2.07). For aJHD cases this was between 0.56 and 0.84% (95% CI 0.11–1.50), for cJHD between 0.28 and 0.42% (95% CI –0.04–0.89) and for PHD 0.09 to 0.14% (95% CI –0.09–0.41).

**Table 2:** Prevalence of JHD in the Netherlands

JHD subtype	Prevalence frequencies *n (%)*	Prevalence < 18 y (PHD) *n (%)*	Prevalence 18–25 y *n*	Prevalence > 25 y *n*
cJHD	3 (0.28–0.42%)	1	1	1
aJHD	6 (0.56–0.84%)	0	1	5
**Total**	**9 (0.84–1.25%)**	**1 (0.09–0.14%)**	**2**	**6**

Columns represent prevalence frequencies (1) in total, (2) of JHD patients that are currently < 18 years of age, referred to as PHD, (3) of JHD patients that are currently between 18 and 25 years of age, and (4) of JHD patients that are currently older than 25 years of age. In addition, number of total cJHD, total aJHD, total JHD and PHD patients are given as a proportion of the total manifest HD population in the Netherlands based on an estimated prevalence of 6:100,000 (left percentage between brackets) or 4:100,000 (right percentage between brackets). n, number of patients; cJHD, childhood-onset JHD; aJHD, adolescent-onset JHD; PHD, pediatric Huntington’s disease.

### Incidence of JHD in the Netherlands

**Fig. 2:**
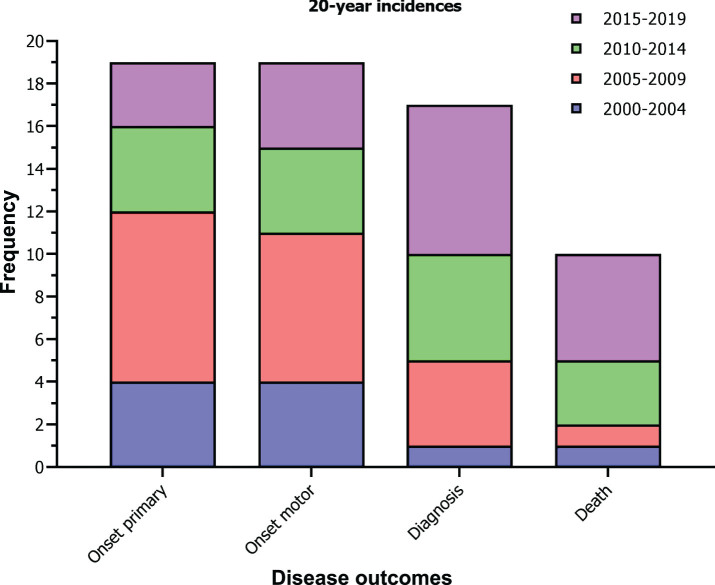
20-year incidences of JHD in the Netherlands. Stacked bar graphs shows the number of JHD cases 1) experiencing the onset of a primary symptom, 2) experiencing the onset of a first motor symptom, 3) receiving a clinical diagnosis of HD, and 4) dying, between 2000 and 2020 and color coded by time frames of 5 years. One-way ANOVA for between timeframe comparisons revealed no statistically significant results (*p*-values > 0.05).

Between 2000 and 2019, a total of 19 JHD cases experienced onset of primary and onset of motor symptoms, 17 JHD cases received a clinical diagnosis and 10 JHD cases died (Fig. 2). In addition, a total of 10 JHD patients were clinically diagnosed≤17 years, therefore diagnosed as PHD patient. Based on this time period, the mean incidence for a clinical JHD diagnosis in relation to the total clinically manifest HD population in the Netherlands (4–6:100,000) was between 0.85 to 1.28 (95% CI –0.96–4.01) per 1000 HD patient years. The mean incidence of a JHD patient dying in the same patient population was between 0.50 to 0.74 (95% CI –0.89–2.85) per 1000 HD patient years. The mean incidence of a clinical diagnosis in a PHD patient, in relation to the general under-aged population in the Netherlands (≤17 years), was 0.14 (95% CI –0.25–0.54) per million person years. These rates imply that, in the situation of the Netherlands, for every 1000 clinically manifest HD patients seen, 1 of them will have a JHD diagnosis and for every 1600, 1 of them dies with JHD. In addition, for every 7.000.000 under-aged individuals in the general population, approximately 1 of them will receive a PHD diagnosis. No statistically significant difference was observed between incidences of the consecutive 5-year time periods.

### Age and disease duration at diagnosis

In the HD-JUNIOR registry, 10 of 22 (45%) JHD cases received a clinical diagnosis of HD before the age of 18 years and 12 of 22 (55%) JHD cases received a clinical diagnosis of HD in adulthood (defined as age≥18). Likewise, In the ENROLL-HD registry 61 of 142 (43%) JHD cases received a clinical diagnosis before the age of 18 years and the other 81 out of 142 (57%) from ENROLL-HD received their clinical diagnosis of HD on adult age. In 5 of 12 JHD cases (42%) from HD-JUNIOR that received a clinical diagnosis at adult age, preclinical genetic testing was performed prior to receiving a clinical HD diagnosis.

**Fig. 3:**
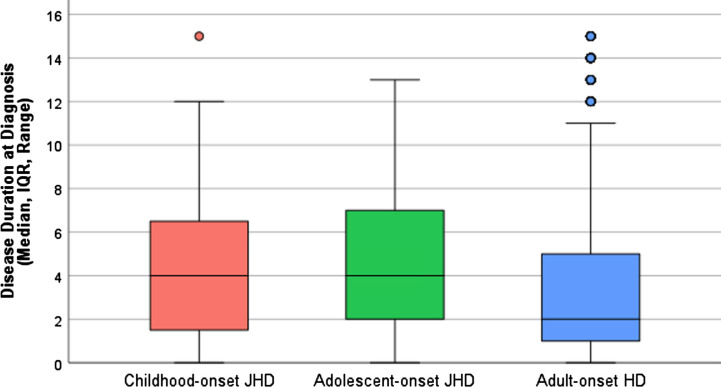
Disease duration at diagnosis in AO-HD subtypes. Boxplots showing the median, IQR, range and outliers of disease duration in years between primary symptom onset and clinical diagnosis of HD in the pooled cJHD (red; *n* = 43), aJHD (green; *n* = 119) and AHD (blue; *n* = 8,808) patient samples. One-way ANOVA of the log10-transformed disease duration at diagnosis revealed a statistically significant mean difference between the aJHD and AHD patient samples (*p* = <0.001).

The median disease duration between primary symptom and a clinical diagnosis of HD was, in cJHD (*n* = 43) 4 years (IQR 1–7), in aJHD (*n* = 119) 4 years (IQR 2–7) and in AHD patients (*n* = 8808) 2 years (IQR 1–5) (Fig. 3). Between AO-HD subtype comparison of the log10-transformed disease duration at diagnosis revealed a statistically significant mean difference between aJHD and AHD patients (mean difference 0.17, 95% CI 0.08–0.25, *p* = <0.001).

### Disease stage markers

In the HD-JUNIOR registry, all cJHD cases (*n* = 5) were functionally compromised at diagnosis (Table 3). Four out of five cases received special primary education and the other one received regular primary education, albeit with difficulty. In addition, chronic home care was needed for all and in one case temporary hospitalization was needed. In aJHD cases of the HD-JUNIOR registry, their functioning at the time of clinical diagnosis was compromised in 7 out of 8 cases (Table 3). In 4 of these 7 compromised aJHD cases, secondary vocational education was discontinued at an early stage and in the other 3 secondary education was discontinued early. In addition, 4 cases received chronic nursery home care and 3 cases received partial care at home or in day care.

**Table 3:** Functional incapacities at diagnosis in JHD subtypes of the HD-JUNIOR registry

Patient	Age Dx (y)	Disease Duration Dx (y)	Education	Care level Dx	Functionally compromised Dx	Specified
JHDc-01-4	8	0.5	Special primary education	Home care (chronic)	Yes	- Needs tricycler
						- Needs help bathing/toileting
						- Needs help with transfers
JHDc-02-X	7	2.5	Primary education (with difficulty)	Home care (chronic)	Yes	- Needs help changing clothes
						- Gave up cycling
JHDc-03-3	10	4	Special primary education	Home care (chronic)	Yes	- Needs walking aids
						- Gave up cycling
						- Needs help maintaining personal hygiene
						- Needs help changing clothes
JHDc-04-4	11	5.5	Special primary education	Home care (chronic) and Inhospitalization (temporary)	Yes	- Change in independence with outdoor activities
						- Needs help changing clothes
JHDc-05-2	15	5.5	Special secondary education	Home care (chronic)	Yes	- Change in independence with outdoor activities
						- Needs walking aids
JHDa-01-1	18	2	Secondary vocational education drop-out	Day care (partial)	Yes	- Gave up cycling
JHDa-02-1	19	3	Secondary education drop-out	Nursery home care (chronic)	Yes	- Change in independence with outdoor activities
						- Gave up job
JHDa-03-X	20	3	Secondary education drop-out	Home care (partial)	Yes	- Cannot find job
						- Needs help with domestic chores
JHDa-04-1	21	3.5	Secondary vocational education drop-out	Home care (partial)	Yes	- Needs help with finances
						- Needs help with domestic chores
JHDa-05-1	21	3.5	Secondary vocational education finished	Independent	No
JHDa-06-2	20	4.5	Secondary vocational education drop-out	Nursery home care (chronic)	Yes	- Difficulty writing
						- Cannot find job
JHDa-07-2	19	6	Secondary education drop out	Nursery home care (chronic)	Yes	- Gave up job
JHDa-08-X	23	11	Secondary vocational education drop-out	Nursery home care (chronic)	Yes	Unknown

Each row displays pseudonymized patient data from the HD-JUNIOR registry in relation to functional incapacities within one year of diagnosis. In the Patient column you find patient characteristics regarding: onset in childhood (JHDc) or in adolescence (JHDa) - case number - CAG-repeat length category (‘1’ for CAGs≥50 and < 60, ‘2’ for CAGs≥60 and < 70, ‘3’ for CAGs≥70 and < 80, ‘4’ for CAGs≥80, ‘X’ in case CAG was unknown). Age at clinical diagnosis and disease duration between primary symptom and clinical diagnosis are given in column 2 and 3. Latest or highest education received at clinical diagnosis are given in the ‘education’ column. Care level at clinical diagnosis in relation to place and partial (is partially independent) or chronic (is largely dependent) care are given in the ‘care level’ column. Dx, at diagnosis.

**Fig. 4:**
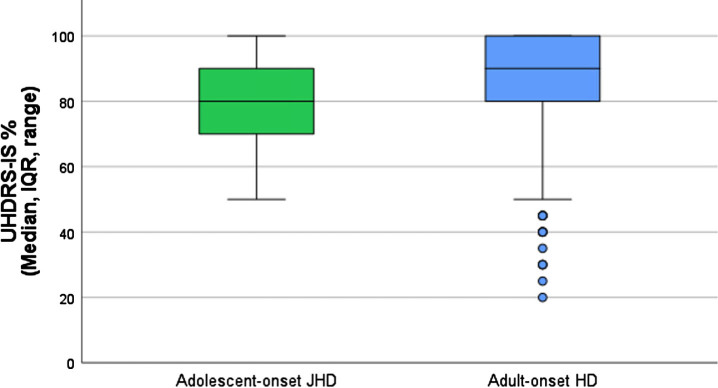
UHDRS-Independent Score at diagnosis in aJHD and AHD subtypes. Boxplots showing the median, IQR, range and outliers of the UHDRS-IS score within one year of clinical diagnosis in the aJHD (green; *n* = 33) and AHD (blue; *n* = 3,186) patient samples of ENROLL-HD. One-way ANOVA of the log10-transformed UHDRS-IS score at diagnosis revealed a statistically significant mean difference between the aJHD and AHD patient samples (*p* = <0.001).

Functional capacity at clinical diagnosis in the ENROLL-HD registry was analyzed by means of the UHDRS-IS score. In four cJHD patients (in whom the UHDRS-IS was completed within one year of receiving a clinical diagnosis of HD), the UHDRS-IS score was twice 90% (‘no physical care needed if difficult tasks are avoided’), once 55% and once 50% (’24-hour supervision appropriate; assistance required for bathing, eating, toileting’). In aJHD patients (*n* = 33), the median UHDRS-IS score at clinical diagnosis was 80% (IQR 70–90%), which refers to ‘pre-disease level of employment/education changes or ends; cannot perform household chores to pre-disease level, may need help with finances’ (Fig. 4). In AHD patients (*n* = 3,186), the median UHDRS-IS at clinical diagnosis was 90% (IQR 80–100%), which refers to ‘no physical care needed if difficult tasks are avoided’ (Fig. 4). Between aJHD and AHD group comparison of the log10-transformed UHDRS-IS at diagnosis, revealed a statistically significant mean difference of –0.04 (95% CI –0.07––0.01, *p* = <0.001). This suggests a lower functional capacity at diagnosis in aJHD patients compared with AHD patients.

**Fig. 5:**
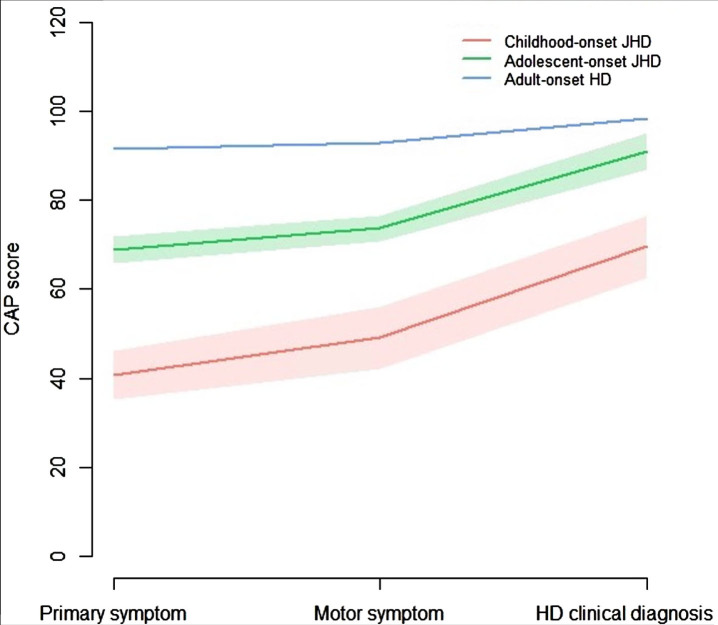
CAP^100^ score over time in AO-HD subtypes. Line graphs showing the mean and 95% CI of the CAP^100^ score at time points: 1) primary symptom onset, 2) motor symptom onset, and 3) clinical diagnosis of HD, in the pooled cJHD (red; *n* = 43), aJHD (green; *n* = 118), and AHD (*n* = 8,808) patient samples. One-way ANOVA for the comparison of the CAP^100^ score at the three different time points between AO-HD subtypes revealed statistically significant differences in means for all time points between all AO-HD subtypes (*p* = <0.001).

As an alternative measure for disease stage, taking into account CAG-repeat length, the CAP^100^ score at age of 1) primary symptom onset, 2) motor symptom onset, and 3) clinical diagnosis was analyzed and compared between AO-HD subtypes of pooled datasets (Fig. 5). The CAP^100^ score progressed from age at primary symptom onset, to age at motor symptom onset, to age at clinical diagnosis of HD in all 3 AO-HD subtypes. The mean CAP^100^ was lowest in the cJHD (*n* = 43), followed by aJHD (*n* = 118) and then AHD (*n* = 8,808) HD-subtype. Intergroup comparisons for the mean CAP^100^ score at the three different time points in AO-HD subtypes were significant by < 0.001 for all comparisons. These outcomes would imply a lower cumulative exposure to the toxic effects of mHTT in cJHD and aJHD patients when compared to AHD patients, and therefore a less severe disease stage at these three fixed time points.

## DISCUSSION

This study reveals the current low prevalence and incidence of JHD and PHD patients in the Netherlands and the limited availability and ability of this population to participate in interventional trials in the near future.

Based on a systematic review and meta-analysis of JHD epidemiology in 2012, the mean proportion of JHD patients as part of the total clinically manifest HD population has been estimated at 4.92% (95% CI 4.07–5.84%).^7^ The last estimate of the proportional prevalence of JHD in the Netherlands, determined in 2002, was 3%.^23^ Yet, one recent study analyzing JHD prevalence in the worldwide HD registry ENROLL-HD, found a substantial lower proportional margin of 1.44%.^8^ Furthermore, the latter study was the first to specify the proportion of JHD patients still under the age of 18 years, referred to as PHD, which was 0.14–0.66%. The results of our study are in line with the latter study and reveal a significantly lower proportional prevalence, between 0.84 and 1.25%, for the JHD population and between 0.09 and 0.14% for the PHD population in the Netherlands, as compared to the estimates from 2002 and 2012. In addition, 20-year incidence rates reveal a stable number of JHD cases over time. This shows that factors such as recognition of the JHD phenotype, treatment options and birth control methods seem to have had no clear influence on the incidence of JHD cases between 2000 and 2020.

Apart from the prevalence and incidence of JHD patients in the Dutch population, our study reveals that JHD patients have a median diagnostic delay of 4 years, and less than half of JHD cases are clinically diagnosed on pediatric age. These JHD cases are labeled as ‘JHD’ in retrospect, already at adult age, and are therefore not available for interventional trials in the PHD population. As has been mentioned before, the design of interventional trials in a PHD population is unrealistic with these small numbers.^12^ Although there are ongoing international efforts to identify as many JHD and PHD cases as possible, it makes us strongly doubt if EMA class waiver removal for pediatric investigation plan in the PHD population outweighs its purpose. In our opinion, the possibility to start compassionate use programs with medical agents tested in the AHD population, should be considered for these rare PHD cases. Moreover, 8 of 9 prevalent JHD cases in the Netherlands are currently above 17 years of age and therefore potentially eligible to participate in interventional trials designed for adult HD cases. Although such JHD cases are often severely affected by the disease (and therefore not comparable to AHD cases), it does make us wonder if alternative trial designs, like for example multiple crossover n-of-1 studies, should be considered.

In the Netherlands, approximately 40% of JHD cases who received a clinical diagnosis on adult age, received their genetic status prior to a clinical diagnosis. This shows that a substantial portion of JHD cases, while experiencing yet unrecognized disease characteristics, are mistakenly counselled for presymptomatic genetic testing by clinical geneticists rather than diagnostic testing by a neurologist. Optimized collaboration and consultation of clinical geneticists and neurologists, in particular in expanded gene risk carriers with a medical history in psychiatric or neurocognitive disease domains, should allow for appropriate counselling for all HD cases in the future.

Part of JHD cases are known to have faster disease progression and a shorter survival when compared to prototypical disease onset in adulthood.^1^ This extremely vulnerable patient population is likely to have a lower ability to participate in the heavy interventional studies that are currently ongoing in the AHD population.^24^ Our study shows that all cJHD patients and most aJHD patients in the Netherlands had severe functional incapacities (HD-ISS stage 3)^19^ when they were clinically diagnosed. This was also reflected by the significantly lower independent scores at diagnosis in aJHD when compared to the adult-onset group in the international Enroll-HD database. This has an impact on the possibility of JHD patients to participate in interventional studies that requires informed consent, long clinical/research visits every few weeks or months and invasive procedures such as venipuncture, lumbar punctures, MRI and other assessments. Furthermore, many UHDRS assessments, like the motor and functional measures, are less suited for the PHD and JHD population.^25,26^ Our study reveals another interesting finding in that respect, by the invalidity of the CAP^100^ score as a predictor of disease progression for the JHD population. The CAP^100^ score is a formula that considers age and CAG-repeat length to predict disease stage and is normalized so that the outcome approximates a score of 100 when an HD patient enters clinical HD-ISS stage 2.^16^ This is grossly in line with the mean CAP^100^ score at diagnosis of 98 that was found in the AHD population of ENROLL-HD. In contrast, the mean CAP^100^ score at diagnosis of 70 in cJHD and 91 in aJHD patients would suggest a lower accumulative exposure to the toxic effects of mHTT in the JHD population, which is highly unlikely given the faster disease progression and shorter survival in this particular population. Lack of fit of this model for CAGs≥50 has already been noted in the original article.^16^ An explanation for these findings could be the non-linear relationship between CAG and age at clinical phenotype that has been found to influence specifically the JHD population, but not AHD population.^3^ Another explanation could be the greater effect of shorter and mutant HTT allele interaction in the JHD population, influencing loss-of-function pathomechanisms.^27^ These outcomes signify the need for adjusted measures to predict disease stage and progression in the JHD population.

Our study has its limitations. In particular the definition of ‘what is a juvenile HD phenotype’ is still under debate and may have had its effects on our prevalence and incidence estimates. Clear international eligibility criteria for a JHD phenotype are needed to ensure consensus in future interventional trials. In addition, it is possible we have missed true JHD cases in our registry due to unrecognized cases (e.g., diagnostic delay; diagnostics that were performed at sites/departments that are unfamiliar with specialized HD care facilities or Enroll-HD) or unwillingness to share medical records for research purposes. Yet, by the synergistic effect of combining genetic and clinical data from multiple sources, our registry is likely to be very conclusive with regard to numbers of cases. Furthermore, due to the limited sample sizes particularly in the functional assessment in the cJHD subtype, the generalizability of these results are limited. However, by working with two different JHD cohorts, these sample sizes are the best that can actually be established in such a rare phenotype. The use of adjusted prospective functional measures as part of standard clinical practice in the JHD population, could help in overcoming the limited generalizability of our study results in the near future.

JHD and PHD are extremely rare and vulnerable HD patient populations, requiring a tailored approach when participating in future interventional trials. Compassionate use programs in PHD cases, alternative trial designs, like multiple cross-over designs, including JHD patients who are aged≥18 in AHD trials, should also be considered. Furthermore, adjusted and validated measures for disease progression in the JHD and PHD population are urgently needed.

## Data Availability

The ENROLL-HD data supporting the findings of this study are openly available at https://studies.enroll-hd.org. The HD-JUNIOR data supporting the findings of this study are available on request from the corresponding author. The data are not publicly available due to privacy and ethical restrictions.
